# Looking Back and Moving Forward: Change and Challenges

**DOI:** 10.1002/jbm4.10556

**Published:** 2021-09-27

**Authors:** Peter R. Ebeling

**Affiliations:** ^1^ Department of Medicine, School of Clinical Sciences Monash University Clayton VIC Australia

Nothing happens unless something is moved. —Albert Einstein

It is the end of the beginning. Nearly 5 years since the birth of *JBMR® Plus*, it is highly appropriate that my final contribution as editor‐in‐chief will be the *JBMR Plus* Early Career Reseacher special issue dedicated to the future leaders of our society. We chose one of the most inspirational figures in our field, Fuller Albright, to grace its cover. Fuller Albright was magical in his immense contributions to the growing science of endocrinology, which he helped found. His astute clinical observations underpinned elegant investigations that identified renal effects of parathyroid hormone, estrogen use in osteoporosis, and vitamin D–resistant rickets, among many others. It is indeed very fitting that his name also honors an ASBMR award for its most meritorious early career researchers as well as the cover of this *JBMR Plus* special issue devoted to their work.

It is challenging to start a new journal. *JBMR Plus* was my second new online‐only open access journal, the first being *Bone Reports*, and this prior experience was invaluable. I also had a lot of help! Help from my four highly competent deputy editors, Drs. Teresita Bellido, Bo Abrahamsen, Fanxin Long, and Luigi Gennari; several talented associate editors, Suzanne Jan de Beur, Stavroula Kousteni, Thorsten Schinke, Xiang‐hong Luo, and most recently, Peggy Cawthon and Emma Clarke; and regional editors, Martina Rauner and Xiang‐hong Luo. The quality of the assembled team was such that three became presidents of either ASBMR or the European Calcified Tissue Society (ECTS) during their *JBMR Plus* tenure! Help from the newly formed *JBMR Plus* Editorial Board and our hard‐working reviewers. Help from the ASBMR Council, two publications directors, Katie Duffy and Kim Murphy, and two executive directors, Ann Elderkin and Doug Fesler, who all enthusiastically supported and believed in ASBMR's new journal. Help from Sarah Dallas and her ASBMR Publications Committee, and help from the experienced and dedicated Wiley publication team, Jessica Downey, Jinnie Wong, and Jane Taylor. Finally, and not least, help from ASBMR members who chose *JBMR Plus* to publish their exciting new work. There would be no *JBMR Plus* without your hard work—thank you to you all! It was a privilege—exciting, humbling, and revelatory—to learn of your latest research, with 40% deciding to submit directly to *JBMR Plus* and a high percentage (currently 22%) accepting our offer to transfer their work from the *JBMR* to *JBMR Plus*.

As editor‐in‐chief, one of my aspirations for *JBMR Plus* was to accelerate the research into bone biology that has underpinned the recent innovative therapeutic advances in bone and mineral research. My senior editorial team allowed us to make *JBMR Plus* a rapidly responsive and innovative home for new discoveries that will ultimately improve global musculoskeletal health by changing clinical care and health policy. We continually strove to drive down turnaround times. Our *JBMR Plus* special issues have highlighted areas we thought were evolving at a rapid rate so were worthy of critical and in‐depth examination. These have included this Early Career Researcher special issue; Vitamin D, in honor of the late and great Dr. Tony Norman; Rare Bone Diseases; and Cancer and Bone. I especially thank my guest editors for their hard work on these special issues: Megan Weivoda and Rachelle Johnson, Roger Bouillon, Eileen Shore and Maurizio Pacifici, and David Roodman, respectively.

An ongoing challenge is to distinguish *JBMR Plus* from a rapidly expanding field of competing online‐only open access journals. The trend for open access is being fostered by institutional and funder mandates to publish in this format. This newly found desire for equity of access allows ASBMR investigators to publish their work so it is immediately available to all. We hope this increased visibility of research published in *JBMR Plus* will also drive an increased recognition of our authors' work. Being affiliated with a specialty scientific society such as ASBMR clearly gives *JBMR Plus* an edge as we move forward to seek recognition by indexing databases. This will be the major challenge in the next 2 years; however, citations are growing at a healthy rate with some articles already being cited >100 times. The future looks bright indeed.

In this unanticipated Olympic year, I am now passing the *JBMR Plus* baton to Dr. Deborah Veis from the Washington University School of Medicine in St. Louis, MO, USA. Deb has the precise credentials to take *JBMR Plus* to the next level, having been a long‐serving associate editor of the *JBMR* under two editors‐in‐chief, Drs. Juliette Compston and Roberto Civitelli. Her background as a bone and mineral clinician and pathologist ideally places her to bring a renewed focus on translational research to the journal. I believe Deb's father was also a scientific editor, so she appears predestined to achieve her role as editor‐in‐chief at a very important time for *JBMR Plus*. Although it is the end of the beginning, the next phase of *JBMR Plus* now commences. With Deb's leadership of her new senior editorial team and your continued support, *JBMR Plus* can only grow stronger and rapidly climb the ladder of endocrinology journals.

In closing, thank you for the privilege of serving as the inaugural editor‐in‐chief of *JBMR Plus*; I have enjoyed the experience immensely, but now we move forward!

### Peer Review

The peer review history for this article is available at https://publons.com/publon/10.1002/jbm4.10556.

